# Post-Ischemic Neurodegeneration of the Hippocampus Resembling Alzheimer’s Disease Proteinopathy

**DOI:** 10.3390/ijms23010306

**Published:** 2021-12-28

**Authors:** Ryszard Pluta, Sławomir Januszewski, Stanisław J. Czuczwar

**Affiliations:** 1 Laboratory of Ischemic and Neurodegenerative Brain Research, Mossakowski Medical Research Institute, Polish Academy of Sciences, Pawińskiego 5 Str., 02-106 Warsaw, Poland; sjanuszewski@imdik.pan.pl; 2 Department of Pathophysiology, Medical University of Lublin, Jaczewskiego 8b Str., 20-090 Lublin, Poland; stanislaw.czuczwar@umlub.pl

**Keywords:** brain ischemia, hippocampus, amyloid, tau protein, presenilin, neuronal death, neurodegeneration, amyloid plaques, neurofibrillary tangles, dementia, genes

## Abstract

In this review, we summarize, inter alia, the protein and gene changes associated with Alzheimer’s disease and their role in post-ischemic hippocampal neurodegeneration. In the hippocampus, studies have revealed dysregulation of the genes for the amyloid protein precursor metabolism and tau protein that is identical in nature to Alzheimer’s disease. Data indicate that amyloid and tau protein, derived from brain tissue and blood due to increased permeability of the blood–brain barrier after ischemia, play a key role in post-ischemic neurodegeneration of the hippocampus, with concomitant development of full-blown dementia. Thus, the knowledge of new neurodegenerative mechanisms that cause neurodegeneration of the hippocampus after ischemia, resembling Alzheimer’s disease proteinopathy, will provide the most important therapeutic development goals to date.

## 1. Introduction

Most studies of post-ischemic brain neurodegeneration have been conducted in rodents. This allows the reconstruction of overlapping pathological mechanisms in the ischemic brain with the simultaneous determination of causal relationships and subsequent consequences. The decisive element in the conduct of ischemic-recirculation brain research in rodents is high homogeneity resulting from inbreeding, availability, low cost and similar organization of the vascular system of the human and rodent brain [1]. The hippocampus is the brain structure of choice for studying the sequelae of ischemic injury for several reasons. First, the hippocampus is a brain structure that exhibits the same neuropathological changes in the ischemic brain and Alzheimer’s disease [2]. Second, the hippocampus is responsible for memory and spatial learning [3]. Third, the CA1 region of the hippocampus is a part of the brain that is highly susceptible to ischemia and changes in Alzheimer’s disease [2]. Following ischemia with recirculation, progressive neurodegeneration in the hippocampus was observed that was dependent on survival following an ischemic episode [4,5,6]. The end result of reversible cerebral ischemia is the enormous death of the sensitive pyramidal neurons of the CA1 region of the hippocampus, with the development of progressive neuroinflammation [4,5,6,7]. In animals surviving 1 year after cerebral ischemia, severe hippocampus atrophy indicates an active, slowly progressive neuropathology that leads to the development of full-blown dementia [3,4,5]. Additionally, it was found that neuropathological phenomena lasted well beyond the acute stage of initial ischemic damage [4,5,6]. The neuropathological picture that is observed in the hippocampus after ischemia shares features with neurodegeneration in Alzheimer’s disease [2,8]. The post-ischemic hippocampus in rodents generates a stereotypical pattern of selective neuronal damage that mimics the hallmarks of sporadic Alzheimer’s disease [2]. The above changes, occurring in sporadic cases of Alzheimer’s disease, suggest that the post-ischemic hippocampus study is a useful in vivo model for elucidating the mechanisms blamed for triggering the development of Alzheimer’s disease [8]. In this review, we present historical and new research related to post-ischemic damage to the hippocampus in terms of proteomic and genomic changes. With regard to exciting new observations of post-ischemic changes in the hippocampus, we present evidence that folding proteins and their genes play an important role in post-ischemic neurodegeneration of the hippocampus along with the development of full-blown dementia.

## 2. Neuropathophysiological Changes in Post-Ischemic Hippocampus

Post-ischemic damage to the hippocampus generates overlapping neuropathophysiological cascades that initiate ischemic damage and death of pyramidal neurons. During the period of ischemia and reperfusion, a huge release of excitatory amino acids and intracellular calcium overload were found [9]. This post-ischemic period is described as an excitotoxic phenomenon due to the unusual release of glutamate [9]. Thus, the discharge of glutamate from the presynaptic terminals and its insufficient reuptake causes an increase in glutamate in the extracellular space of the hippocampus [9]. The consequence of the above-mentioned phenomenon is extreme stimulation of glutamate receptors, which leads to a huge influx of calcium to neuronal cytoplasm through calcium channels [9]. Calcium is also released from intracellular organelles into the cytoplasm of neurons. Accumulated intracellular calcium activates enzymes that are essential for the survival or death of neurons. For example, phospholipases, nitric oxide synthase, proteases and endonucleases are activated by calcium, and the ultimate result of this action is damage to the nucleus, cytoplasmic membranes and organelles. The pathological pathways mentioned above are the factor that directly causes damage or death of pyramidal neurons following an ischemia-reperfusion episode in the hippocampus. Characteristically, acute death of pyramidal neurons, immediately following hippocampus ischemia, occurs through necrosis and delayed death occurs through a programmed process called apoptosis. Necrosis occurs due to the loss of energy and osmotic homeostasis of the pyramidal neurons and involves a huge amount of pyramidal neurons in the hippocampus. Ischemic neurons swell as a result of absorbing excess water, which causes the disruption of cell membranes, and this causes the content of neurons to leak into the adjacent tissue [10]. DNA cleavage in necrotic pyramidal neurons is in the late phase, which occurs through a mechanism requiring serine proteases [11]. Necrosis is caused by the rapid depletion of energy in neurons and the uptake of glucose during hippocampal ischemia. Apoptosis was observed in CA1 pyramidal neurons 4 days after ischemia-reperfusion injury [12]. The two most important processes trigger apoptosis of neurons after ischemia: receptor-mediated or mitochondrial-mediated apoptosis. In the mitochondrial apoptotic pathway, cytochrome c is released into the cytoplasm in sensitive neurons following ischemic damage to the hippocampus [13,14]. Following ischemia-reperfusion injury to the hippocampus, caspase 3 also plays a role in the death of pyramidal neurons [15,16,17,18,19]. It is also proposed to link autophagy and mitophagy with apoptosis [16,17,18,19]. Different caspases can cleave poly (ADP-ribose) polymerase-1 post-ischemia. This action causes DNA fragmentation, which initiates the death of the pyramidal neurons via apoptosis. In addition, excessive activation of poly (ADP-ribose) polymerase-1 initiates energy deficiency, which leads to the death of neuronal cells. The death receptor family contributes to the activation of the death mechanism during apoptosis [20]. The activated receptors stimulate procaspase 8. Caspase 8 stimulates caspase 3, which cleaves poly (ADP-ribose) polymerase-1 and launches caspase-activated DNAse, which causes DNA damage and fragmentation, and ultimately the death of neurons [15]. Delayed death of pyramidal neurons following hippocampal ischemia is induced by apoptotic mechanisms [14]. Following hippocampal ischemia, another type of pyramidal neuronal cell death occurs, called necroptosis [21]. In this phenomenon, features of necrosis and apoptosis after ischemic damage to the hippocampus are present in the same pyramidal neuron [22]. Another process of neuronal death is called autophagy-programmed cell death [23]. In this phenomenon, autolysosomes and autophagosomes are present in dying neuronal cells [23]. Autophagy is thought to protect neurons from death through apoptosis, but also acts as a trigger for neuronal death [24]. According to the first option, the aforementioned lysosomal degradation of the cytoplasm’s own structures provides the neuron with substrates for energy production and protein synthesis. The second option is blamed on autophagy-programmed neuronal cell death [16,17,18,19]. Recent evidence points towards autophagy as a key player in post-ischemic hippocampal neurodegeneration [16,17,18,19,24,25].

## 3. Neurodegenerative Changes in Post-Ischemic Hippocampus

### 3.1. Patterns of Neuronal Pathology

With reference to the above data, neuropathological changes in the hippocampus in various experimental models of ischemic brain injury are presented [26,27,28,29]. In these models, necrotic death of pyramidal neurons in the CA1 region of the hippocampus was observed in the acute phase following ischemia. Then, delayed neuronal death was observed through a programmed mechanism called apoptosis [4,5,26,27,30,31,32]. Damage to the pyramidal neurons has also been reported in the CA1 region of the hippocampus after occlusion of the middle cerebral artery [33,34]. Progression of death of pyramidal neuronal cells was recorded between 2 and 7 days post-ischemia [26]. Three minutes of cerebral ischemia in gerbils and 10 min in rats were sufficient to induce characteristic changes in the hippocampus [4,5,26,27,30,31,32]. Twenty minutes of cerebral ischemia in rats causes complete loss of pyramidal neurons in the CA1 area of the hippocampus and damage to neurons in the striatum and cortex [27]. Prolongation of post-ischemic survival causes damage to pyramidal neurons in areas of the hippocampus with non-selective sensitivity to ischemia [4,5]. Two years after cerebral ischemia, various types of neuropathological changes were found in pyramidal neuronal cells of non-selective sensitivity [4,6]. The first type was chronic and the second type was acute neuronal damage and appeared in the CA2, CA3 and CA4 regions of the hippocampus [4,5]. In support of the above changes, an increased co-location of Fluoro Jade C and NeuN (neurons undergoing apoptosis) signals was observed after ischemia in the CA3 sector, indicating an increased number of pyramidal neurons undergoing apoptotic death even 2 years post-ischemia [6]. In this area, the loss of pyramidal neurons was mixed up with acute and chronic neuronal degeneration, and the changes during this time period were intense and diffuse [4,5]. The macroscopic and microscopic examinations carried out at that time showed the atrophy of the hippocampus [4]. In addition, ipsilateral hippocampal atrophy has been associated with impairment of long-term memory after ischemic stroke in patients [35,36].

### 3.2. Synaptic Alterations 

The synaptic integrity of the hippocampus is essential for learning and memory [37]. Following local cerebral ischemia in the rat hippocampus, a decrease in the concentration of postsynaptic synaptophysin and a densification of the protein 95 was found [38,39]. Additionally, in ischemic rats, ultrastructural synaptic changes in the CA1 area of the hippocampus were noted [37]. Other investigations have shown that ischemia induces synaptic autophagy that is associated with the death of pyramidal neurons in the CA1 subfield of the hippocampus [40,41]. Experimental reversible cerebral ischemia provides evidence of isolated and persistent synaptic failure in the hippocampus [37,42]. A reduction in the excitatory synaptic transmission in the post-ischemic state was observed in the CA1 region of the hippocampus [9]. Ischemia induces an increase in intracellular calcium and enhances the activity of calpains in pyramidal neuronal cells, and calpain target proteins are present at glutamatergic and GABAergic synapses. In the brain after ischemia, calpains cleave pre- and postsynaptic proteins. The cleavage of calpain-related protein contributes to the loss of pyramidal neurons in the hippocampus following ischemia [43]. Following cerebral ischemia, loss of the pyramidal neurons in the CA1 region is apparent, along with a decline in acetylcholine levels in the hippocampus [44]. This suggests that the death of pyramidal neurons is also due to the inability of the hippocampus to transmit excitatory and cholinergic transmission to neurons [9,44,45]. 

## 4. Blood–Brain Barrier Permeability in Post-Ischemic Hippocampus

Following hippocampal ischemia, extravasated horseradish peroxidase, a marker of blood–brain barrier permeability, was observed as diffuse, mild, random, focal and speckled foci of staining around the blood–brain barrier vessels [5,46,47,48,49]. The permeability of the blood–brain barrier was limited to bifurcation and branching of vessels in the post-ischemic hippocampus [47]. Leakage involved arterioles, capillaries, venules and veins. Intensive perivascular staining of various fragments of the amyloid precursor protein was observed around the blood–brain barrier vessels following hippocampal ischemia [5,47]. Numerous and profuse deposits encompassed or adhered to the blood–brain barrier vessels, spreading outward in a multifocal manner into adjacent tissue [5,47]. Deposits of various parts of the amyloid protein precursor around the blood–brain barrier vessels were dominant in the CA1 and CA4 regions [5,47]. Perivascular deposits of various parts of the amyloid protein precursor and horseradish peroxidase took the same forms in the hippocampus after ischemia [5,47]. Staining around the blood–brain barrier vessels of horseradish peroxidase and various parts of the amyloid protein precursor confirmed that these molecules cross the blood–brain barrier [5,47]. These indicated disruptions of the blood–brain barrier for the above molecules after ischemia. The above observation supported examination of the leakage of human amyloid across the post-ischemic blood–brain barrier in rats [47,50]. Blood–brain barrier permeability for human amyloid involved arterioles, capillaries, venules and veins. Endothelial cells, pericyte cells and astrocytes were filled with amyloid. These changes were located predominantly in the hippocampus and brain cortex [50]. There is also evidence that, as with amyloid, the tau protein crosses the blood–brain barrier from peripheral circulation to the brain following brain ischemia [51,52]. The observed permeability of the blood–brain barrier was long-term as it was still present 12 months after ischemia and was associated with the continued death of pyramidal neurons and an increased number of activated neuroglial cells in all areas of the hippocampus [4,5,6,7,47].

## 5. Neuroinflammation in Post-Ischemic Hippocampus

Strong microglia and astrocyte responses have been observed in the areas of selective changes of neuronal cells in the hippocampus [6,7,30,31,32,53,54,55,56,57]. A strong cytokine response in astrocytes was observed in the CA1 region of the ischemic hippocampus [57]. These data show that the increase in neuroinflammatory factors in astrocytes is directly related to the selective susceptibility of neuronal cells to ischemia-reperfusion brain injury [57,58]. These results suggest that neurons in sensitive sectors in the ischemic hippocampus are targets for interleukin-1β generated by astrocytes. This observation can be explained by the increased expression of the neuronal interleukin-1 receptor [58]. Interleukin-1β was shown to play a key role not only in cellular changes but also in the development of brain edema following ischemia-reperfusion injury [59]. Long-term generation of neuroinflammatory factors by the ischemic hippocampus activates the self-sustaining cycle that leads to the ischemic pathology characteristic of neurodegeneration in Alzheimer’s disease. In cerebral ischemia and Alzheimer’s disease, interleukin-1 is the major player that guides neuronal cells to the amyloidogenic metabolism of the amyloid protein precursor and the release of neuroinflammatory mediators [60]. These types of pathways lead to abnormalities in the activity of neuronal cells and ultimately to their death. The death of neuronal cells results from neuroinflammatory pathogens that induce neuronal changes. Activating microglial cells causes further strengthening and self-spreading of the neuroinflammatory cycle. The release of neuroinflammatory factors by microglia is also promoted by β-amyloid peptide [61]. In the hippocampus, the activation of glial cells precedes neuronal damage and lasts long after an episode of ischemia, i.e., up to two years [6]. Brain atrophy develops in areas where neuronal cells have disappeared and the spider web has been completely destroyed. 

In a long-term post-ischemic survival study, the chronic effect of cerebral ischemia on the neuroinflammatory response, generated by microglia and astrocytes, was demonstrated in the rat hippocampus even 2 years after injury [6]. Microglia and astrocyte activation was revealed in the CA1 and CA3 regions [6]. In particular, in ischemia-sensitive areas of the hippocampus, microglia and astrocytes simultaneously showed significant activation, while in ischemia-resistant areas of the hippocampus, only astrocytes were active, suggesting that less intense neuroinflammation occurs in areas of the hippocampus that are resistant to ischemia [6].

## 6. Amyloid Accumulation in Post-Ischemic Hippocampus

In rodents with survival up to 0.5 years post-ischemia, intracellular and extracellular accumulation of all parts of the amyloid protein precursor in the hippocampus was found. [30,62,63,64,65,66,67,68,69]. Accumulation of various parts of the amyloid protein precursor was present in neuronal and neuroglial cells [30,66,68,70,71,72]. Half a year after ischemia, only the C-terminal of the amyloid protein precursor and the amyloid has been documented [4,30,67,73]. Data indicate that astrocytes, which accumulate large amounts of different parts of the amyloid protein precursor, are involved in the formation of glial scar [30,68,72]. In addition, reactive astrocytes with excessive amyloid accumulation may be involved in repairing the hippocampus tissue after ischemia, which ultimately leads to the death of astrocytes [30,62,68,74]. The extracellular amyloid accumulation showed the features of diffuse amyloid plaques [4,30,62,67,68]. It was shown that diffuse amyloid plaques do not go away but have the ability to transform into senile plaques roughly one year post-ischemia [75]. Amyloid deposition in neuronal cells and astrocytes is a sign of pathological metabolism of the amyloid protein precursor during ischemic neurodegeneration of the hippocampus [31,32,62,65,72]. The data clearly show that the accumulation of amyloid after ischemia in the hippocampus is responsible for the secondary degenerative processes that cause progressive death of pyramidal neurons, which further influences the post-ischemic outcome [3,4,66,67,73,76,77]. 

Diffuse and senile amyloid plaques have also been found in the hippocampus in patients with a history of cerebral ischemia [78,79,80,81,82]. According to a study performed after cerebral ischemia in humans, the accumulation of β-amyloid peptide 1–42 and 1–40 was observed in the hippocampus [81]. Increased deposition of various amyloids contributes to the progression of ischemic neurodegenerative processes and ultimately to the development of Alzheimer’s disease dementia. In addition, clinical trials have shown an increase in the level of amyloid in the blood in patients with a history of cerebral ischemia [83,84,85]. Increased plasma amyloid in these patients was found to be negatively correlated with neurological outcomes after ischemic brain injury [84].

The expression of the *amyloid protein precursor* (*APP*) gene in the CA1 subfield of the hippocampus was decreased 2 days after ischemia and increased above the control values between 7 and 30 days (Table 1) [86]. The *β-secretase* (*BACE1*) and *presenilin 1* and *2* (*PSEN1* and *2*) genes were upregulated within 2–7 days and decreased 30 days after ischemia (Table 1) [86]. 

In the CA3 area of the hippocampus, 2 and 30 days post-ischemia, *APP* gene expression fluctuated around control values (Table 1) [87]. In contrast, 7 days post-ischemia, *APP* gene expression was above control values (Table 1) [87]. Expression of the *BACE1* gene in the CA3 region was below the control values within 2–7 days, while 30 days after ischemia, it was above the control values (Table 1) [87]. Expression of the *PSEN1* gene was above control values within 2–7 days, and fluctuated around the control values 30 days after ischemia (Table 1) [87]. Post-ischemic *PSEN2* expression fluctuated around the control values within 2 days, decreased on day 7 and was above the control values on day 30 (Table 1) [87].

## 7. Tau Protein Modification in Post-Ischemic Hippocampus

After experimental cerebral ischemia, strong accumulation of tau protein in pyramidal neurons in the hippocampus was observed [69,88]. Tau protein deposition was also increased in post-ischemic neuroglial cells [89,90]. The above data mean that pyramidal neurons are characterized by an increased accumulation of the tau protein, which indicates the neuropathological processes taking place in these cells. Another study indicates that the modified tau protein influences the transport of amyloid from the neuron body through axons and dendrites, resulting in amyloid accumulation in the neuronal body [91]. In addition, studies have shown that post-ischemia, the hyperphosphorylated tau protein dominates neuronal cells and accompanies apoptosis [89,90,92,93,94]. The above observations indicate that the apoptosis of pyramidal neuronal cells after hippocampal ischemia is directly related to the hyperphosphorylated tau protein. Further evidence demonstrates that ischemia influences the formation of paired helical filaments [95], neurofibrillary tangle-like [92,93,94] and finally neurofibrillary tangles [96,97]. Tau protein conversion into neurofibrillary tangles is a pathological neuronal response to an ischemic episode of the hippocampus [69,89,90,92,93,94,95,96,98,99,100]. Moreover, in clinical trials, increased serum tau protein was found in humans with a history of cerebral ischemia, and these data indicate an advanced stage of post-ischemic changes in neuronal cells [101,102,103,104,105,106,107]. The above observations are the basis for the development of post-ischemic dementia with the Alzheimer’s disease phenotype. 

Two–seven days after ischemia in the CA1 area of the hippocampus, the expression of the *MAPT* gene was higher than the control values (Table 1) [108]. However, on day 30 post-ischemia, *MAPT* gene expression was lower than control values (Table 1) [108]. In the CA3 region, the expression of the *MAPT* gene fluctuated around the control values within 2 days, but 7–30 days after ischemia, the expression was higher than in the control group (Table 1) [87]. 

## 8. Metals in Post-Ischemic Hippocampus

Another factor involved in the neurodegeneration of the hippocampus post-ischemia is the disturbance of zinc and iron homeostasis [109,110,111]. Zinc levels remain constant in the adult animal’s hippocampus and are involved in learning and memory [112]. An ischemic episode disrupts the neuronal homeostasis of zinc, contributing to the death of pyramidal neuronal cells in the hippocampus [113,114]. After reversible cerebral ischemia, zinc accumulates, particularly in degenerated neurons in the CA1 area of the hippocampus [109,114]. During ischemia, high concentrations of zinc released from a subset of glutamatergic terminals promote zinc translocation and accumulation in sensitive postsynaptic neurons, resulting in neurotoxic death of postsynaptic neurons [109]. Toxic zinc influx may be a key mechanism underlying the selective death of neurons in the hippocampus following reversible ischemia. 

Glutathione is the most abundant thiol in cells and plays a key role in defending cells against disturbed zinc homeostasis. In neuronal cells, the level of glutathione is regulated by the excitatory amino acid carrier 1, which transports cysteine, necessary for the synthesis of glutathione. Evidence suggests that deletion of the excitatory amino acid carrier 1 gene exacerbates post-ischemic damage to the hippocampus through impaired zinc homeostasis [115], and diurnal fluctuations in excitatory amino acid carrier 1 levels affect the susceptibility of pyramidal neurons in the hippocampus to ischemic death [116]. 

Zinc increases the toxicity of amyloid, and sequestration of zinc into amyloid deposits leads to the loss of essential zinc for the proper functioning of synapses [117]. Amyloid oligomers interact with intracellular zinc, resulting in zinc deficiency, and zinc deficiency causes neurodegeneration and cognitive decline such as learning and memory impairment [109,118]. Zinc influences amyloid neurotoxicity by stabilizing amyloid fibrils. An in vitro study showed that zinc induces the rapid and extensive aggregation of synthetic amyloid, which can act as a seed factor in the formation of amyloid plaques [119]. In support of these observations, high levels of zinc were found in amyloid plaques in murine models of Alzheimer’s disease [119].

Disruption of zinc homeostasis leads to the formation of neurofibrillary tangles composed of hyperphosphorylated tau protein [119]. Zinc is involved in the hyperphosphorylation of the tau protein through a direct or indirect mechanism [119]. These two independent mechanisms of action have different effects on the toxicity of the tau protein. The direct interaction between the tau protein and zinc has been found to play an important role in tau protein toxicity. Zinc is also believed to be a catalyst that accelerates the aggregation of the tau protein and, at the same time, promotes the formation of tau protein oligomers [119].

Elevated hippocampal iron levels and iron-mediated oxidative stress also play a role in hippocampus ischemic pathology [120,121]. Iron deposition and overload are important triggers of ferroptosis. Ferroptosis is a new form of iron-dependent cell death and differs from other types of cell death such as apoptosis, necrosis and autophagy in biochemical, morphological and genetic aspects [111]. Ferroptosis is characterized by iron accumulation, lipid peroxidation, production of reactive oxygen species, reduced glutathione levels, suppression of glutathione peroxidase 4 and altered expression of many genes [111,122].

It was found that the level of amyloid aggregation influences the iron redox cycle and consequently leads to the release of free radicals via the Fenton reaction [119]. Iron dysregulation also affects oxidative stress in tauopathies [119]. Iron is involved in the hyperphosphorylation of the tau protein through a direct or indirect mechanism [119] and binds to the tau protein, causing irreversible structural changes in it [119]. The result of this interaction is the aggregation of the tau protein and development of oxidative stress through the Fenton reaction, which has an impact on the state of cell damage [119].

## 9. Dementia and Post-Ischemic Hippocampus

Behavioral changes occur as a result of ischemic damage to the pyramidal neurons in the hippocampus [3,123,124,125]. In animals, post-ischemic locomotor hyperactivity [126,127], similar to that in patients with Alzheimer’s disease, has been observed. Hyperactivity was associated with the loss of pyramidal neuronal cells in the hippocampus [126]. A longer time of ischemia and, consequently, a longer duration of locomotor hyperactivity, were directly related to the increased number of pyramidal neurons deaths and inflammation in the hippocampus [5,6,7,128]. Habitual disorders after ischemia were found, manifested by prolonged examination time [129]. Cerebral ischemia causes a deficiency in reference and working memory [3]. Post-ischemic hippocampal damage in animals slowly leads to spatial memory deficits during survival [3,130]. The progression of cognitive deficits was recorded each time throughout the entire period of recirculation in the brain [3,130]. In addition, recurrent and reversible cerebral ischemic episodes in gerbils showed persistent locomotor hyperactivity, reduced anxiety, and persistent cognitive deficits [131]. The behavioral deficits mentioned above were associated with massive hippocampal atrophy [4,30,31,32,73,76,77,132] and death of pyramidal neurons mainly in the CA1 area of the hippocampus [4,5,77,131]. Learning and memory deficits are progressive and of permanent duration [3,128]. 

A dangerous pattern of ischemic brain injury in patients is the gradual and progressive expansion of post-ischemic dementia [35,133,134,135,136], which is associated with severe neurological disability [137] and hippocampal atrophy [35,36]. Worldwide, post-stroke dementia affects 5% to 50% patients depending on diagnostic criteria, population demography and geographic location [137]. Epidemiological studies have shown that the incidence of dementia in people who survive 3 months after ischemia is approximately nine times higher than in healthy subjects [137]. Patients who survived ischemia develop dementia in about 22% by the end of 4 years during the follow-up period [138]. The incidence of dementia after recurrent ischemic stroke is estimated at 33% [137]. 

## 10. Conclusions

**Figure 1:**
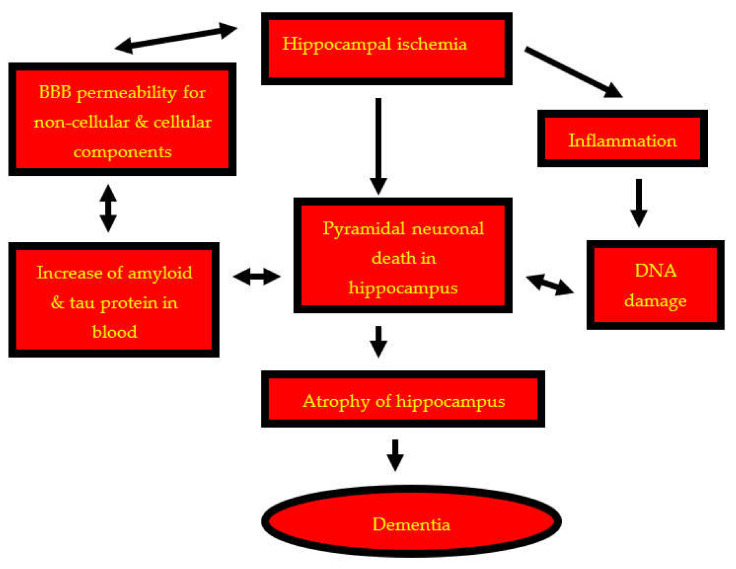
Development of pyramidal neuron death, atrophy of hippocampus and finally dementia. BBB—blood–brain barrier.

The presented data indicate that post-ischemic neurodegeneration of the hippocampus is associated with the acute and chronic death of pyramidal neurons, the generation of amyloid and modified tau protein, and additionally, chronic leakage of amyloid and tau protein through the ischemic blood–brain barrier from systemic circulation to the brain parenchyma (Figure 1). The passage of amyloid and tau protein across the blood–brain barrier after ischemia was demonstrated using macro- (horseradish peroxidase) and micromolecular markers (human β-amyloid 1–42 peptide) [47]. Intravenous administration of horseradish peroxidase is routinely used as a marker for post-ischemic blood–brain barrier permeability; in addition, human β-amyloid peptide and tau protein have also been used [47,50,51]. Immediately following hippocampal ischemia, numerous extracellular diffuse, benign, random, focal and patchy foci of horseradish peroxidase, β-amyloid peptide and tau protein around and near the blood–brain barrier vessels were observed [1,46,47,48,50,62,139,140]. The post-ischemic hippocampus was dominated by extracellular accumulation of horseradish peroxidase and amyloid. The above deposits were labeled with specific antibodies [47,50]. The permeability of the blood–brain barrier in the hippocampus was dominant in vascular branching and bifurcations of the blood–brain barrier vessels after ischemia. The numerous and profuse deposits encompassed or adhered to the blood–brain barrier vessels, spreading multifocally to adjacent tissue. Perivascular amyloid and horseradish peroxidase deposits retained the same form in the post-ischemic hippocampus [47]. This indicates a disruption of the blood–brain barrier for the above molecules after ischemia. There is also evidence that, as with amyloid, the tau protein crosses the blood–brain barrier [51] from the systemic circulation to the hippocampus tissue following ischemia. Additionally, using an electron microscope, platelets on the outside of blood–brain barrier vessels after an ischemic episode of the brain were visualized [1,139,140]. The observed permeability of the blood–brain barrier was still present 1 year after ischemia and was associated with the development of diffuse amyloid plaques, modified tau protein and progressive death of pyramidal neurons, and an increased number of activated neuroglial cells in areas of the hippocampus that are sensitive and insensitive to ischemia (Figure 1) [4,5,6,7,46,48,49,139,140]. The development of neuroinflammation in all sectors of the hippocampus, even 2 years post-ischemia, was observed (Figure 1) [6,7]. It should be emphasized that following cerebral ischemia, progressive death of neurons develops in all sectors of the hippocampus with full-blown dementia (Figure 1). It has been shown that neurodegenerative processes in the hippocampus are chronic. The underlying message of this presentation is that post-ischemic neuropathological processes in the hippocampus represent a creeping progression from the initial ischemic changes and death of pyramidal neurons to the well-established production and extravasation of amyloid and tau protein across the post-ischemic blood–brain barrier to the brain from systemic circulation. These processes, which are associated with chronic neuronal death in insensitive areas of the hippocampus, culminate in the formation of amyloid plaques and neurofibrillary tangles, and in the end, resemble Alzheimer’s disease proteinopathy with full-blown dementia (Figure 1) [139,140]. The presented phenomena after hippocampal ischemia have a remarkable parallel with Alzheimer’s disease.

## Figures and Tables

**Table 1:** Expression of genes associated with Alzheimer’s disease in CA1 and CA3 sectors in post-ischemic hippocampus.

	Genes	*APP*	*BACE1*	*PSEN1*	*PSEN2*	*MAPT*
Survival						
CA1 sector						
2 days		↓	↑↑	↑	↑↑	↑↑
7 days		↑	↑	↑	↑	↑
30 days		↑	↓	↓	↓	↓
CA3 sector						
2 days		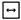	↓	↑	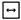	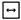
7 days		↑	↓	↑	↓	↑
30 days		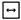	↑	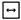	↑	↑

Expression: ↑ increase; ↑↑ increase; ↓ decrease; 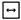 oscillation around control values. Genes: *APP*—amyloid protein precursor; *BACE1*—β-secretase; *PSEN1*—presenilin 1; *PSEN2*—presenilin 2; *MAPT*—tau protein.

## Data Availability

Not applicable.
